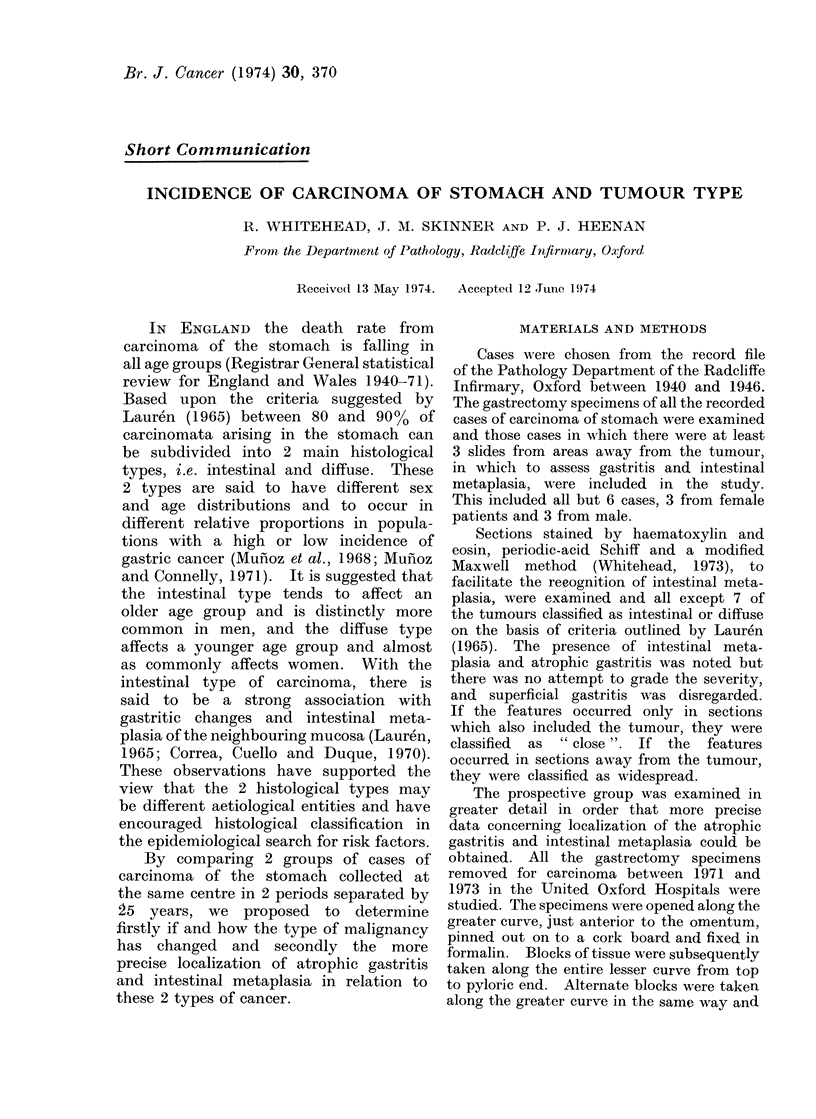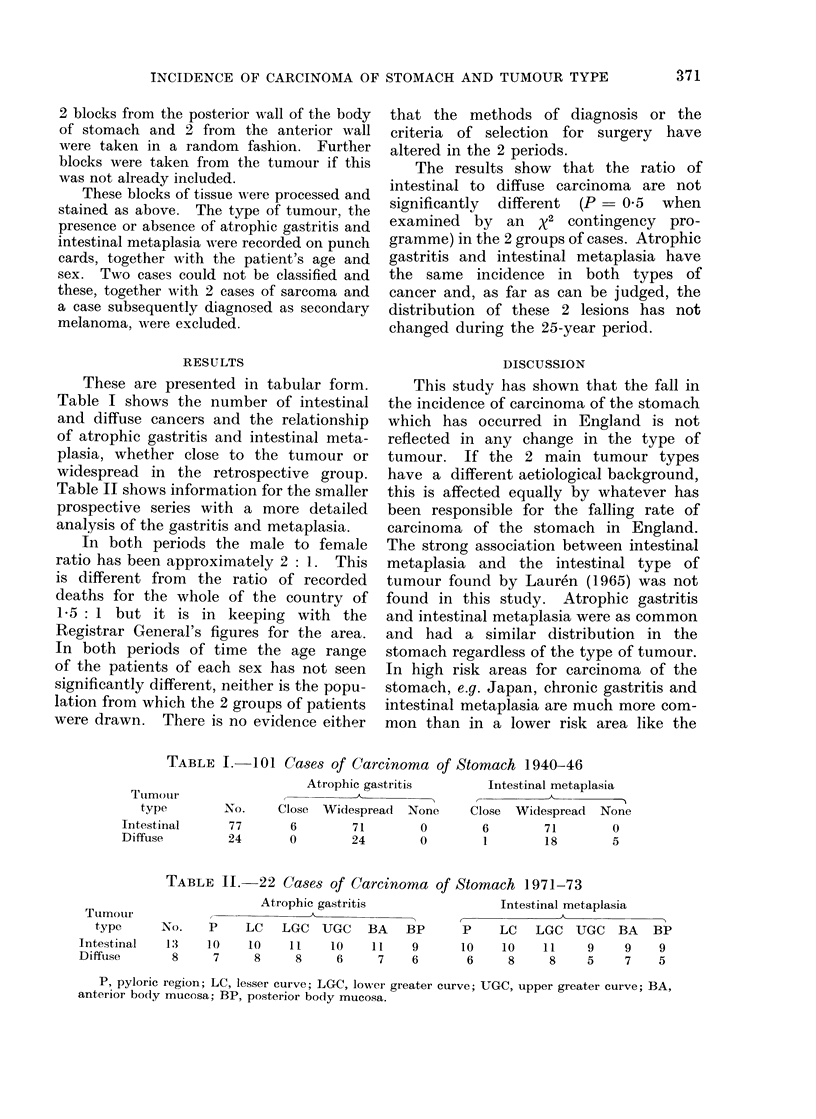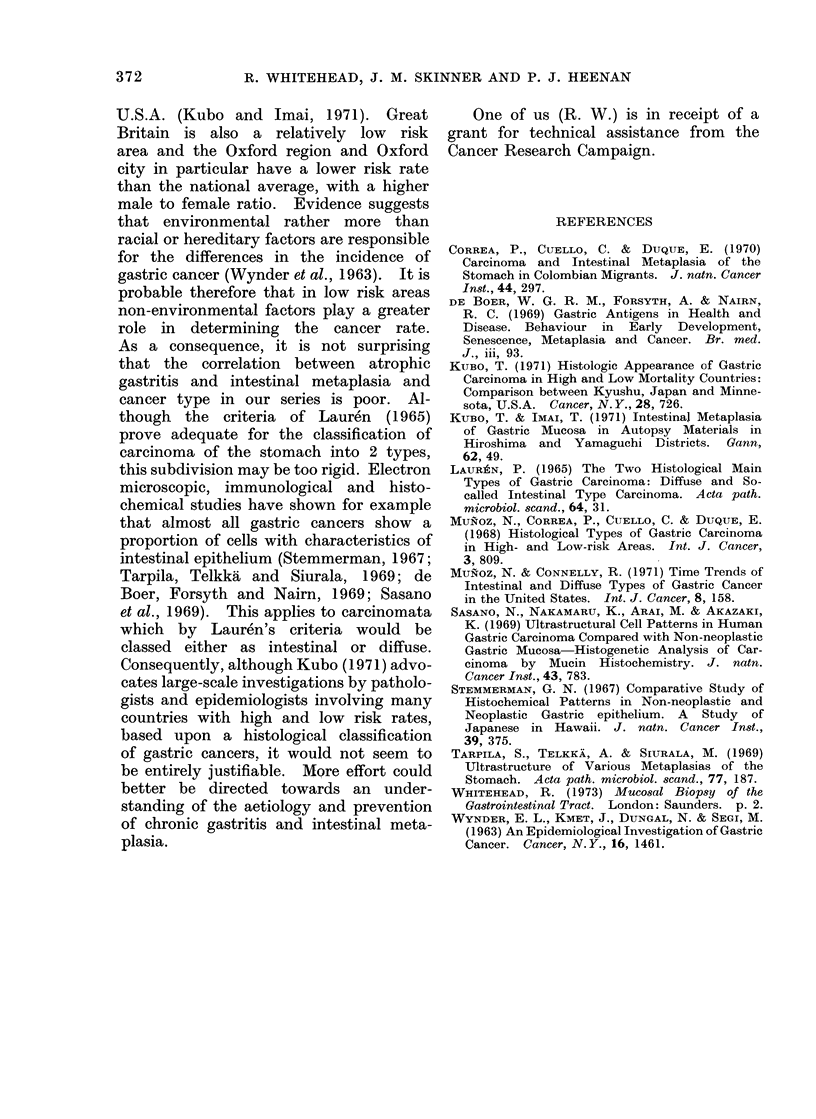# Incidence of carcinoma of stomach and tumour type.

**DOI:** 10.1038/bjc.1974.207

**Published:** 1974-10

**Authors:** R. Whitehead, J. M. Skinner, P. J. Heenan


*Br. J. Cancer* (1974) **30**, 370

***Short Communication***

### INCIDENCE OF CARCINOMA OF STOMACH AND TUMOUR TYPE

R. WHITEHEAD, J. M. SKINNER AND P. J. HEENAN

*From the Department of Pathology, Radcliffe Infirmary, Oxford*

Received 13 May 1974. Accepted 12 June 1974

IN ENGLAND the death rate from carcinoma of the stomach is falling in all age groups (Registrar General statistical review for England and Wales 1940–71). Based upon the criteria suggested by Laurén (1965) between 80 and 90% of carcinomata arising in the stomach can be subdivided into 2 main histological types, *i.e.* intestinal and diffuse. These 2 types are said to have different sex and age distributions and to occur in different relative proportions in populations with a high or low incidence of gastric cancer (Muñoz *et al.*, 1968; Muñoz and Connelly, 1971). It is suggested that the intestinal type tends to affect an older age group and is distinctly more common in men, and the diffuse type affects a younger age group and almost as commonly affects women. With the intestinal type of carcinoma, there is said to be a strong association with gastritic changes and intestinal metaplasia of the neighbouring mucosa (Laurén, 1965; Correa, Cuello and Duque, 1970). These observations have supported the view that the 2 histological types may be different aetiological entities and have encouraged histological classification in the epidemiological search for risk factors.

By comparing 2 groups of cases of carcinoma of the stomach collected at the same centre in 2 periods separated by 25 years, we proposed to determine firstly if and how the type of malignancy has changed and secondly the more precise localization of atrophic gastritis and intestinal metaplasia in relation to these 2 types of cancer.

### MATERIALS AND METHODS

Cases were chosen from the record file of the Pathology Department of the Radcliffe Infirmary, Oxford between 1940 and 1946. The gastrectomy specimens of all the recorded cases of carcinoma of stomach were examined and those cases in which there were at least 3 slides from areas away from the tumour, in which to assess gastritis and intestinal metaplasia, were included in the study. This included all but 6 cases, 3 from female patients and 3 from male.

Sections stained by haematoxylin and eosin, periodic-acid Schiff and a modified Maxwell method (Whitehead, 1973), to facilitate the recognition of intestinal metaplasia, were examined and all except 7 of the tumours classified as intestinal or diffuse on the basis of criteria outlined by Laurén (1965). The presence of intestinal metaplasia and atrophic gastritis was noted but there was no attempt to grade the severity, and superficial gastritis was disregarded. If the features occurred only in sections which also included the tumour, they were classified as " close ". If the features occurred in sections away from the tumour, they were classified as widespread.

The prospective group was examined in greater detail in order that more precise data concerning localization of the atrophic gastritis and intestinal metaplasia could be obtained. All the gastrectomy specimens removed for carcinoma between 1971 and 1973 in the United Oxford Hospitals were studied. The specimens were opened along the greater curve, just anterior to the omentum, pinned out on to a cork board and fixed in formalin. Blocks of tissue were subsequently taken along the entire lesser curve from top to pyloric end. Alternate blocks were taken along the greater curve in the same way and

2 blocks from the posterior wall of the body of stomach and 2 from the anterior wall were taken in a random fashion. Further blocks were taken from the tumour if this was not already included.

These blocks of tissue were processed and stained as above. The type of tumour, the presence or absence of atrophic gastritis and intestinal metaplasia were recorded on punch cards, together with the patient's age and sex. Two cases could not be classified and these, together with 2 cases of sarcoma and a case subsequently diagnosed as secondary melanoma, were excluded.

### RESULTS

These are presented in tabular form. Table I shows the number of intestinal and diffuse cancers and the relationship of atrophic gastritis and intestinal metaplasia, whether close to the tumour or widespread in the retrospective group. Table II shows information for the smaller prospective series with a more detailed analysis of the gastritis and metaplasia.

In both periods the male to female ratio has been approximately 2 : 1. This is different from the ratio of recorded deaths for the whole of the country of 1·5 : 1 but it is in keeping with the Registrar General's figures for the area. In both periods of time the age range of the patients of each sex has not seen significantly different, neither is the population from which the 2 groups of patients were drawn. There is no evidence either that the methods of diagnosis or the criteria of selection for surgery have altered in the 2 periods.

The results show that the ratio of intestinal to diffuse carcinoma are not significantly different ($P = 0{\cdot}5$ when examined by an $\chi^2$ contingency programme) in the 2 groups of cases. Atrophic gastritis and intestinal metaplasia have the same incidence in both types of cancer and, as far as can be judged, the distribution of these 2 lesions has not changed during the 25-year period.

### DISCUSSION

This study has shown that the fall in the incidence of carcinoma of the stomach which has occurred in England is not reflected in any change in the type of tumour. If the 2 main tumour types have a different aetiological background, this is affected equally by whatever has been responsible for the falling rate of carcinoma of the stomach in England. The strong association between intestinal metaplasia and the intestinal type of tumour found by Laurén (1965) was not found in this study. Atrophic gastritis and intestinal metaplasia were as common and had a similar distribution in the stomach regardless of the type of tumour. In high risk areas for carcinoma of the stomach, *e.g.* Japan, chronic gastritis and intestinal metaplasia are much more common than in a lower risk area like the

TABLE I.—101 *Cases of Carcinoma of Stomach* 1940–46

| Tumour type | No. | Atrophic gastritis | | | Intestinal metaplasia | | |
|---|---|---|---|---|---|---|---|
| | | Close | Widespread | None | Close | Widespread | None |
| Intestinal | 77 | 6 | 71 | 0 | 6 | 71 | 0 |
| Diffuse | 24 | 0 | 24 | 0 | 1 | 18 | 5 |

TABLE II.—22 *Cases of Carcinoma of Stomach* 1971–73

| Tumour type | No. | Atrophic gastritis | | | | | | Intestinal metaplasia | | | | | |
|---|---|---|---|---|---|---|---|---|---|---|---|---|---|
| | | P | LC | LGC | UGC | BA | BP | P | LC | LGC | UGC | BA | BP |
| Intestinal | 13 | 10 | 10 | 11 | 10 | 11 | 9 | 10 | 10 | 11 | 9 | 9 | 9 |
| Diffuse | 8 | 7 | 8 | 8 | 6 | 7 | 6 | 6 | 8 | 8 | 5 | 7 | 5 |

P, pyloric region; LC, lesser curve; LGC, lower greater curve; UGC, upper greater curve; BA, anterior body mucosa; BP, posterior body mucosa.

U.S.A. (Kubo and Imai, 1971). Great Britain is also a relatively low risk area and the Oxford region and Oxford city in particular have a lower risk rate than the national average, with a higher male to female ratio. Evidence suggests that environmental rather more than racial or hereditary factors are responsible for the differences in the incidence of gastric cancer (Wynder *et al.*, 1963). It is probable therefore that in low risk areas non-environmental factors play a greater role in determining the cancer rate. As a consequence, it is not surprising that the correlation between atrophic gastritis and intestinal metaplasia and cancer type in our series is poor. Although the criteria of Laurén (1965) prove adequate for the classification of carcinoma of the stomach into 2 types, this subdivision may be too rigid. Electron microscopic, immunological and histochemical studies have shown for example that almost all gastric cancers show a proportion of cells with characteristics of intestinal epithelium (Stemmerman, 1967; Tarpila, Telkkä and Siurala, 1969; de Boer, Forsyth and Nairn, 1969; Sasano *et al.*, 1969). This applies to carcinomata which by Laurén's criteria would be classed either as intestinal or diffuse. Consequently, although Kubo (1971) advocates large-scale investigations by pathologists and epidemiologists involving many countries with high and low risk rates, based upon a histological classification of gastric cancers, it would not seem to be entirely justifiable. More effort could better be directed towards an understanding of the aetiology and prevention of chronic gastritis and intestinal metaplasia.

One of us (R. W.) is in receipt of a grant for technical assistance from the Cancer Research Campaign.

### REFERENCES

Correa, P., Cuello, C. & Duque, E. (1970) Carcinoma and Intestinal Metaplasia of the Stomach in Colombian Migrants. *J. natn. Cancer Inst.*, **44**, 297.

de Boer, W. G. R. M., Forsyth, A. & Nairn, R. C. (1969) Gastric Antigens in Health and Disease. Behaviour in Early Development, Senescence, Metaplasia and Cancer. *Br. med. J.*, iii, 93.

Kubo, T. (1971) Histologic Appearance of Gastric Carcinoma in High and Low Mortality Countries: Comparison between Kyushu, Japan and Minnesota, U.S.A. *Cancer, N.Y.*, **28**, 726.

Kubo, T. & Imai, T. (1971) Intestinal Metaplasia of Gastric Mucosa in Autopsy Materials in Hiroshima and Yamaguchi Districts. *Gann*, **62**, 49.

Laurén, P. (1965) The Two Histological Main Types of Gastric Carcinoma: Diffuse and So-called Intestinal Type Carcinoma. *Acta path. microbiol. scand.*, **64**, 31.

Muñoz, N., Correa, P., Cuello, C. & Duque, E. (1968) Histological Types of Gastric Carcinoma in High- and Low-risk Areas. *Int. J. Cancer*, **3**, 809.

Muñoz, N. & Connelly, R. (1971) Time Trends of Intestinal and Diffuse Types of Gastric Cancer in the United States. *Int. J. Cancer*, **8**, 158.

Sasano, N., Nakamaru, K., Arai, M. & Akazaki, K. (1969) Ultrastructural Cell Patterns in Human Gastric Carcinoma Compared with Non-neoplastic Gastric Mucosa—Histogenetic Analysis of Carcinoma by Mucin Histochemistry. *J. natn. Cancer Inst.*, **43**, 783.

Stemmerman, G. N. (1967) Comparative Study of Histochemical Patterns in Non-neoplastic and Neoplastic Gastric epithelium. A Study of Japanese in Hawaii. *J. natn. Cancer Inst.*, **39**, 375.

Tarpila, S., Telkkä, A. & Siurala, M. (1969) Ultrastructure of Various Metaplasias of the Stomach. *Acta path. microbiol. scand.*, **77**, 187.

Whitehead, R. (1973) *Mucosal Biopsy of the Gastrointestinal Tract.* London: Saunders. p. 2.

Wynder, E. L., Kmet, J., Dungal, N. & Segi, M. (1963) An Epidemiological Investigation of Gastric Cancer. *Cancer, N.Y.*, **16**, 1461.